# Supplementary material for: The activity of the aryl hydrocarbon receptor in T cells tunes the gut microenvironment to sustain autoimmunity and neuroinflammation
Source: PLoS Biol. 2023 Feb 14;21(2):e3002000. doi: 10.1371/journal.pbio.3002000 (PMC9928083; doi:10.1371/journal.pbio.3002000)
Supplement: S1 Fig — (A) No genotype differences in EAE incidence from the experiment shown in Fig 1A. (B) No differences in EAE clinical score or (C) incidence in cohoused male mice (males; n = 7–8; N = 1; Mann–Whitney U Test [p = 0.5680]). (D) No genotype differences in EAE incidence from the experiment shown in Fig 1D. (E) Separately housed male Cd4creAhrfl/fl mice recover from EAE, while Ahrfl/fl mice maintain paralysis. (Males; n = 7–8/group; N = 1; Mann–Whitney U Test on total scores reported in legend [p = 0.0010] and on single days reported on plot.) (F) No difference in incidence of cohoused male mice. Raw data can be found in Supporting information (S1 Data). (PDF) [file pbio.3002000.s001.pdf]

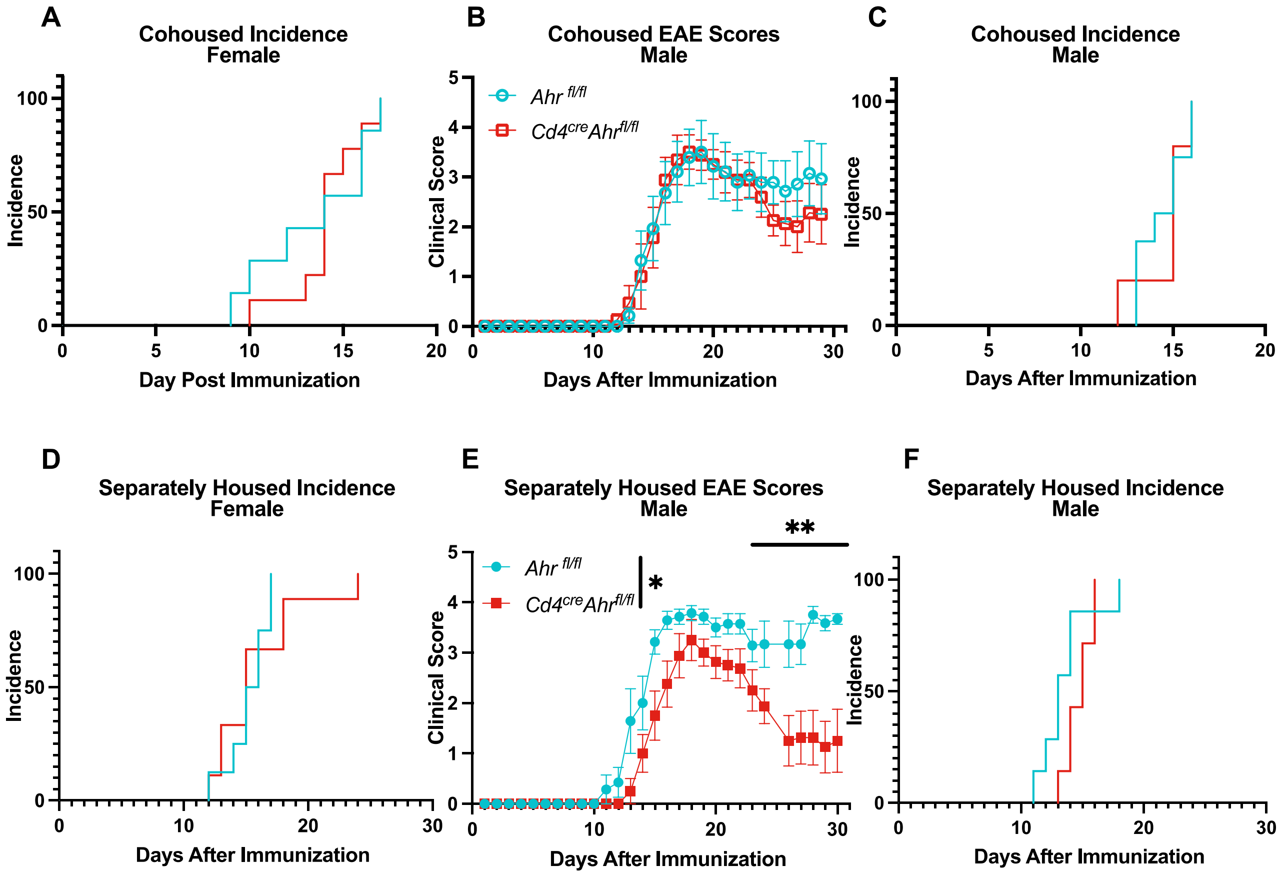

S1 Fig. Separately housed *Cd4^{cre}Ahr$^{fl/fl}$* recover from EAE phase. (**A**) No genotype differences in EAE incidence from the experiment shown in **Fig 1A**. (**B**) No differences in EAE score or (**C**) incidence in cohoused male mice (Males; n= 7-8; N=1; Mann-Whitney U Test [p=0.5680]) (**D**) No genotype differences in EAE incidence from the experiment shown in **Fig 1D**. (**E**) Separately housed male *Cd4^{cre}Ahr$^{fl/fl}$* mice recover from EAE while *Ahr$^{fl/fl}$* mice maintain paralysis. (Males; n=7-8/group; N=1; Mann-Whitney U Test on total scores reported in legend [p=0.0010] and on single days reported on plot) (**F**) No difference in incidence of cohoused male mice.
