# Supplementary material for: The activity of the aryl hydrocarbon receptor in T cells tunes the gut microenvironment to sustain autoimmunity and neuroinflammation
Source: PLoS Biol. 2023 Feb 14;21(2):e3002000. doi: 10.1371/journal.pbio.3002000 (PMC9928083; doi:10.1371/journal.pbio.3002000)
Supplement: S2 Fig — Naïve T cells were isolated from separately housed at weaning animals and differentiated toward TH1, TH2, TH17, and Treg in vitro. Transcription factor expression was quantified by flow cytometry. (A) Quantification of Tbet+ cells differentiated to TH1 cell type and stimulated for 24 h with either anti-CD3 (anti-CD3) or a combination of anti-CD28 and anti-CD3 (anti-CD28) (n = 4 mice/group; N = 1 experiment; Ordinary one-way ANOVA [p = 0.2927]). (B) Quantification of GATA3+ cells differentiated to TH2 cell type and stimulated for 24 h with either anti-CD3 (anti-CD3) or a combination of anti-CD28 and anti-CD3 (anti-CD28) (n = 4 mice/group; N = 1 experiment; Ordinary one-way ANOVA [p = 0.0076] followed by Tukey’s post hoc). (C) Quantification of RORγt+ cells differentiated to TH17 cell type and stimulated for 24 h with either anti-CD3 or anti-CD28 (n = 4 mice/group; N = 1 experiment; Ordinary one-way ANOVA [p = 0.0163] followed by Tukey’s post hoc). (D) Quantification of FoxP3+ cells differentiated to Treg cell type and stimulated for 24 h with either anti-CD3 or anti-CD28 (n = 4 mice/group; N = 1 experiment; Ordinary one-way ANOVA [p = 0.8801]). (E) IFNγ ELISA on supernatant of differentiated TH1 cells stimulated for 24 h with either anti-CD3 (anti-CD3) or a combination of anti-CD28 and anti-CD3 (anti-CD28) (n = 4 mice/group; N = 1 experiment; Ordinary one-way ANOVA [p = 0.8639]). (F) IL-4 ELISA on supernatant of differentiated TH2 cells stimulated for 24 h with either anti-CD3 or anti-CD28 (n = 4 mice/group; N = 1 experiment; Ordinary one-way ANOVA [p = 0.0064] followed by Tukey’s post hoc). (G) IL-17a ELISA on supernatant of differentiated TH17 cells stimulated for 24 h with either anti-CD3 or anti-CD28 (n = 4 mice/group; N = 1 experiment; Ordinary one-way ANOVA [p = 0.4826]). Raw data can be found in Supporting information (S1 Data). (PDF) [file pbio.3002000.s002.pdf]

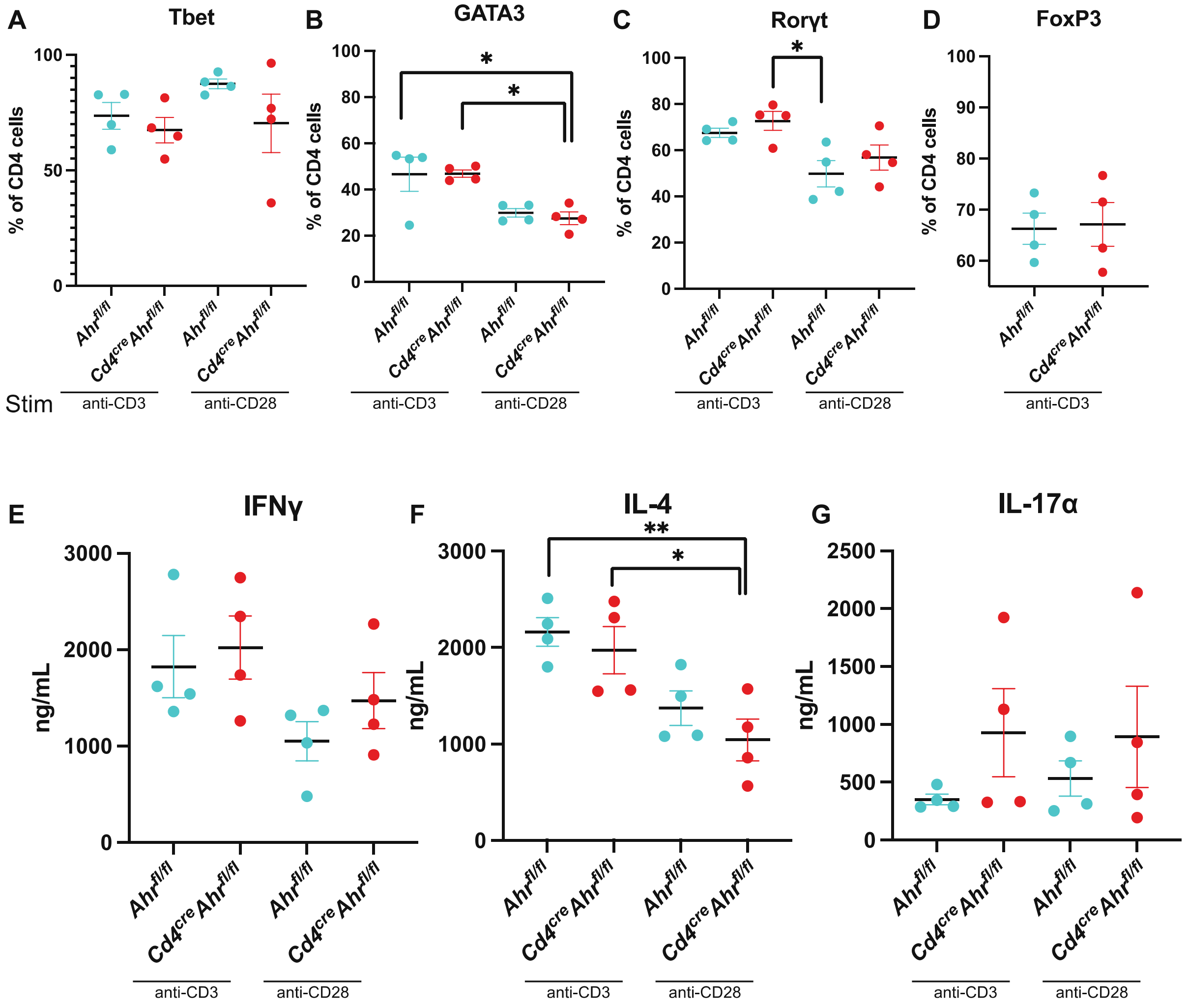

S2 Fig. CD4+ cells from separately housed *Cd4^{cre}Ahr$^{fl/fl}$* have no change in differentiation capacity or cytokine production. (**A**) Flow cytometric quantification of Tbet+ cells differentiated to T_H1 cell type and stimulated for 24 hours with either anti-CD3 or anti-CD28 (n=4 mice/group; N=1 experiment; Ordinary one-way ANOVA [p=0.2927]). (**B**) Flow cytometric quantification of GATA3+ cells differentiated to T_H2 cell type and stimulated for 24 hours with either anti-CD3 or anti-CD28 (n=4 mice/group; N=1 experiments; Ordinary one-way ANOVA [p=0.0076] followed by Tukey's Post-Hoc). (**C**) Flow cytometric quantification of RORγt+ cells differentiated to T_H17 cell type and stimulated for 24 hours with either anti-CD3 or anti-CD28 (n=4 mice/group; N=1 experiment; Ordinary one-way ANOVA [p=0.0163] followed by Tukey's Post-Hoc). (**D**) Flow cytometric quantification of FoxP3+ cells differentiated to T_{reg} cell type and stimulated for 24 hours with either anti-CD3 or anti-CD28 (n=4 mice/group; N=1 experiment; Ordinary one-way ANOVA [p=0.8801]). (**E**) IFNγ ELISA on supernatant of differentiated T_H1 cells stimulated for 24 hours with either anti-CD3 or anti-CD28 (n=4 mice/group; N=1 experiment; Ordinary one-way ANOVA [p=0.8639]). (**F**) IL-4 ELISA on supernatant of differentiated T_H2 cells stimulated for 24 hours with either anti-CD3 or anti-CD28 (n=4 mice/group; N=1 experiment; Ordinary one-way ANOVA [p=0.0064] followed by Tukey's Post-Hoc). (**G**) IL-17a ELISA on supernatant of differentiated T_H17 cells stimulated for 24 hours with either anti-CD3 or anti-CD28 (n=4 mice/group; N=1 experiment; Ordinary one-way ANOVA [p=0.4826]).
