# Supplementary material for: The activity of the aryl hydrocarbon receptor in T cells tunes the gut microenvironment to sustain autoimmunity and neuroinflammation
Source: PLoS Biol. 2023 Feb 14;21(2):e3002000. doi: 10.1371/journal.pbio.3002000 (PMC9928083; doi:10.1371/journal.pbio.3002000)
Supplement: S3 Fig — (A) Gene expression analysis (qPCR array) performed on spinal cord RNA prepared from separately housed Cd4creAhrfl/fl mice and Ahrfl/fl littermate controls at the peak of disease (day 16) shows increased pro-apoptotic transcripts and decreased anti-apoptotic transcript (cDNA prepared from 2 spinal cords were pooled for each sample). (B) Gating strategy for Annexin V+ apoptotic and necrotic cells shown in show in Figs 4 and S3C and S3D. (C) Quantification of total dead cells in separately housed TH17 differentiated cells (n = 3 mice/group; N = 1 experiment; unpaired t test [p = 0.5130]). (D) T cells differentiated to TH1, TH2, or Treg in vitro from separately housed Cd4creAhrfl/fl mice have higher expression of the apoptotic marker Annexin V after 24 h of CD3 stimulation. (n = 3/group; N = 1 experiment; unpaired t tests [p = 0.4276, 0.0078, 0.0442]). Raw data can be found in Supporting information (S1 Data). (PDF) [file pbio.3002000.s003.pdf]

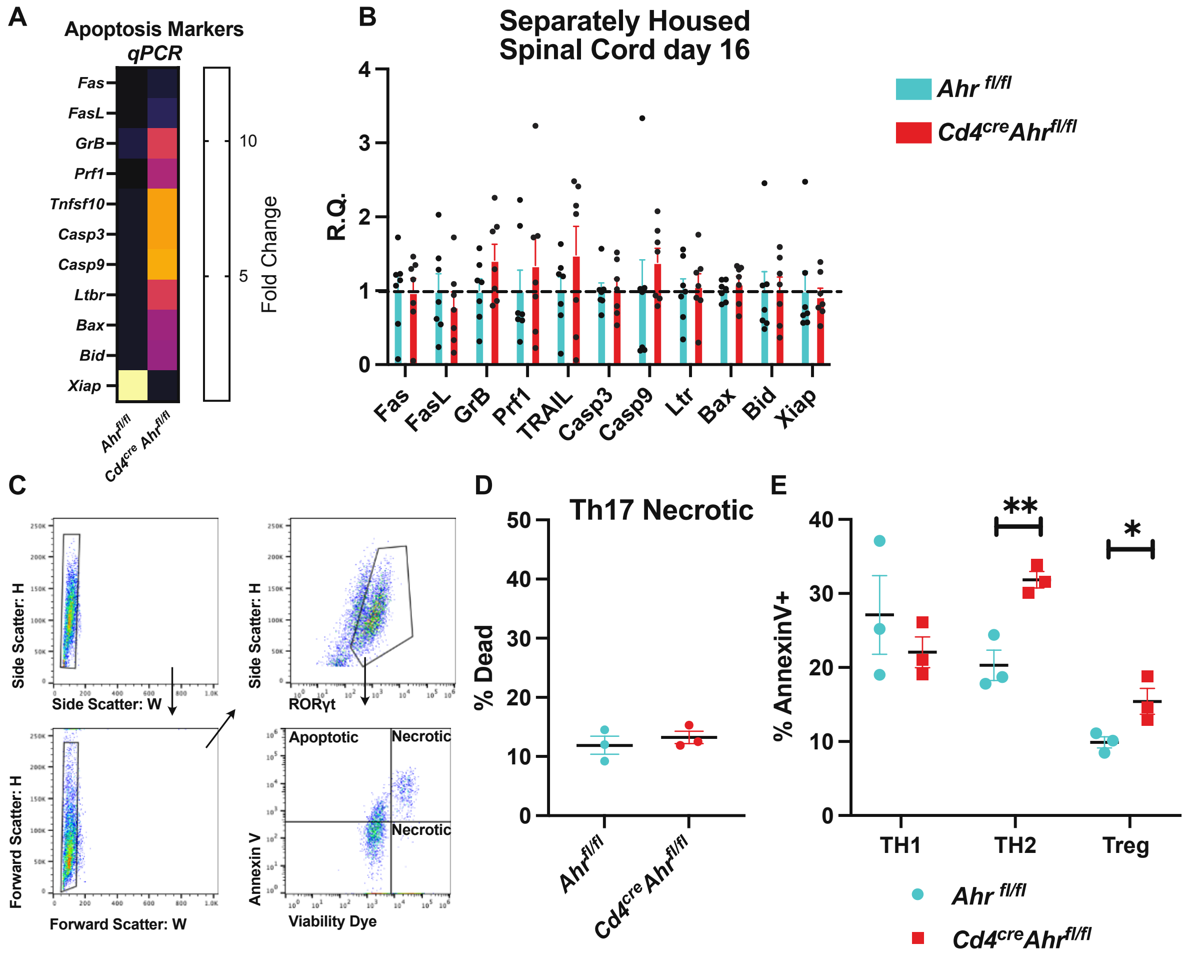

S3 Fig. Apoptosis in T cells of separately housed *$Cd4^{cre}Ahr^{fl/fl}$* mice. (**A**) Gene expression analysis (qPCR array) performed on spinal cord RNAs prepared from separately housed *$Cd4^{cre}Ahr^{fl/fl}$* mice and *$Ahr^{fl/fl}$* littermate controls at the peak of disease (Day 16) shows increased pro-apoptotic transcripts and decreased anti-apoptotic transcript. (**B**)qPCR of spinal cord homogenate at peak of disease for the apoptosis related markers shown in (A) (Males and Females; n=6-7 mice/group, N=2 experiments). (**C**) Gating strategy for Annexin 5+ apoptotic and necrotic cells shown in show in Fig 4 and S3D-E Fig. (**D**) Quantification of total dead cells in separately housed TH17 skewed cells (n=3 mice/group; N=1 experiment; Unpaired T Test [p=0.5130]). (**E**) T cells skewed to TH1, TH2, or T_{reg} *in vitro* from separately housed *$Cd4^{cre}Ahr^{fl/fl}$* mice have higher levels of the apoptotic marker Annexin 5 after 24 hours of CD3 stimulation. (n=3/group; N=1 experiment; Unpaired T tests [p=0.4276, 0.0078, 0.0442])
