# Supplementary material for: The activity of the aryl hydrocarbon receptor in T cells tunes the gut microenvironment to sustain autoimmunity and neuroinflammation
Source: PLoS Biol. 2023 Feb 14;21(2):e3002000. doi: 10.1371/journal.pbio.3002000 (PMC9928083; doi:10.1371/journal.pbio.3002000)
Supplement: S4 Fig — (A) PCA plot and (B) microbiome composition at the genus level obtained from 16S sequencing performed on feces collected from cohoused animals. (Males; n = 6/group) (C) PCA plot and (D) microbiome composition at the genus level obtained from 16S sequencing performed on feces collected from separately housed animals. (Males and females; n = 10/group) (E) PCA plot and (F) microbiome composition at the genus level obtained from 16S sequencing performed on cecum collected from separately housed animals. (Males and females; n = 12/group). Raw data are available in the NCBI Gene Expression Omnibus repository accession number GSE200440; https://www.ncbi.nlm.nih.gov/geo/query/acc.cgi?acc=GSE200440. (PDF) [file pbio.3002000.s004.pdf]

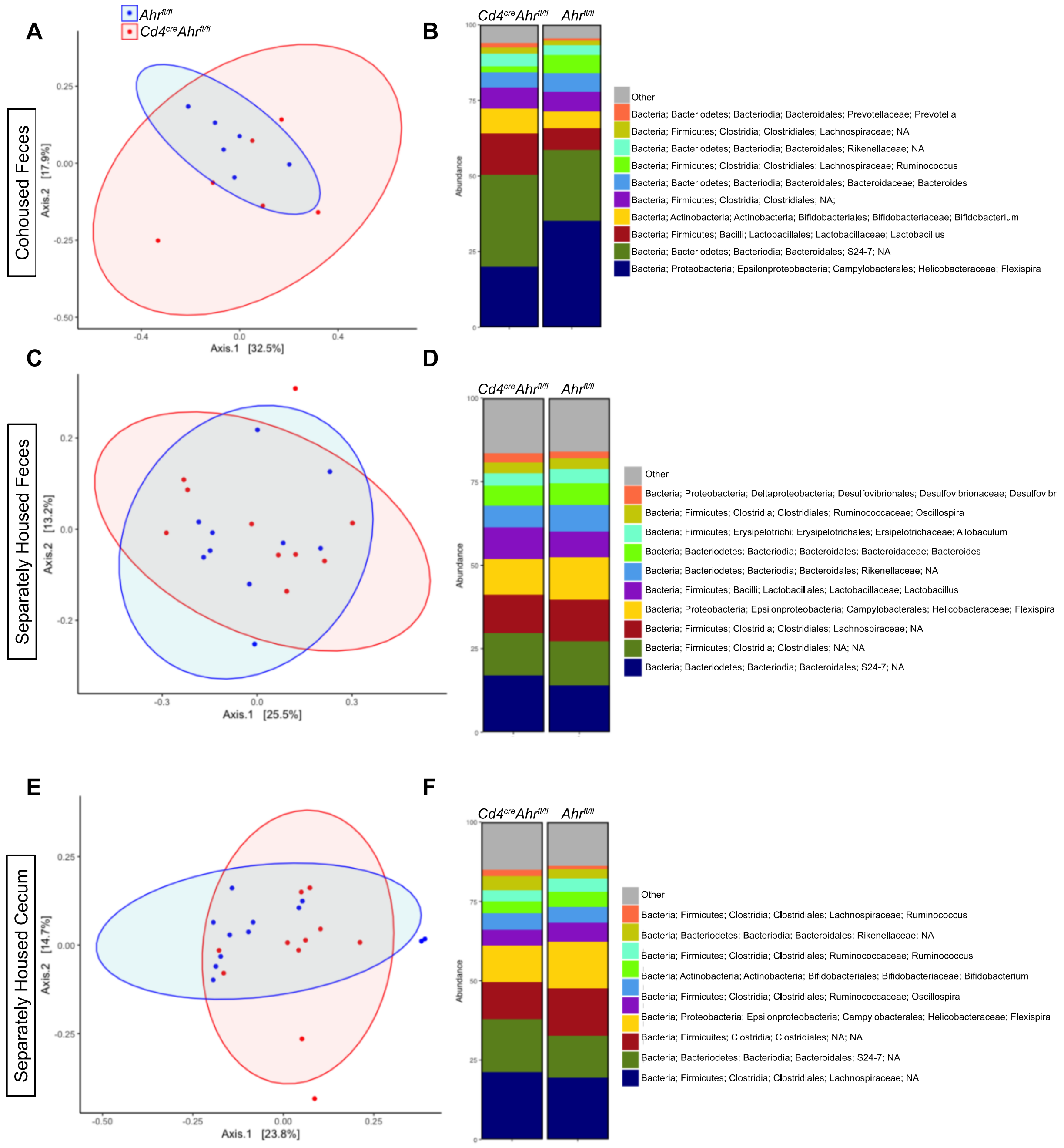

S4 Fig. Only subtle microbiota population changes in *Cd4^{cre}Ahr$^{fl/fl}$* mice (**A**) PCA plot and (**B**) microbiome composition at the genus level obtained from 16S sequencing performed on feces collected from cohoused animals. (Males; n=6/group) (**C**) PCA plot and (**D**) microbiome composition at the genus level obtained from 16S sequencing performed on feces collected from separately housed animals. (Males and Females; n= 10/group) (**E**) PCA plot and (**F**) microbiome composition at the genus level obtained from 16S sequencing performed on cecum collected from separately housed animals. (Males and Females; n=12/group)
