# Supplementary material for: The activity of the aryl hydrocarbon receptor in T cells tunes the gut microenvironment to sustain autoimmunity and neuroinflammation
Source: PLoS Biol. 2023 Feb 14;21(2):e3002000. doi: 10.1371/journal.pbio.3002000 (PMC9928083; doi:10.1371/journal.pbio.3002000)
Supplement: Attachment — Submitted filename: Point by point response to reviewers.pdf [file pbio.3002000.s008.pdf]

Dear Dr. Jauregui and Reviewers,

We would like to thank you for your insightful reviews of our manuscript. In this resubmission, we have addressed the concerns and comments. We have tracked out changes to clearly indicate the edits that we have made. We have responded to the specific concerns from each reviewer point-by-point and detailed the changes below along with the lines in the manuscript where the changes can be found.

Reviewer #1:

Comment #1: One of the main claims are that the remission phenotype seen in CD4ΔAhr mice is microbiota-dependent. In order to support the first claim of microbiota-dependence, a fecal microbiota transplant (FMT) from CD4ΔAhr mice into unrelated C57BL/6 mice that transfers the remission phenotype and elevated T cell apoptosis in the CNS would be most convincing. The inclusion of a section called "Microbiome Transfer" in the Methods section suggests the authors are familiar with this technique and may have completed the experiment already.
Answer: This experiment is now presented in Figure 5.

Comment #2: If an FMT isn't feasible, data demonstrating that the remission in cohoused Ahrfl/fl mice have a metabolite pool that resembles CD4ΔAhr mice and apoptotic cells in the CNS could be used as additional evidence that the phenotype is transmissible via the microbiome.
Answer: FMT was performed, and the results are presented in figure 6D-F showing that microbiota transfer in Bl6 mice can recapitulate the phenotype observed in Figure 1B.

Comment #3: The other main claim is that the remission phenotype is due to impaired T cell fitness downstream of altered pools of microbial-derived metabolites. Given the identification of candidate metabolites (taurocholic acid, isovaleric acid) that influence T cell viability in vitro, an in vivo experiment demonstrating the ability of one or both of these metabolites (or the filtered cecal metabolite pool, depending on experimental feasibility) to induce remission associated with increased T cell apoptosis in the CNS would be welcome.
Answer: Thank you for this suggestion, we have completed this experiment using oral treatment with taurocholic acid (Figure 6 G-I) and shown that this treatment is protective in EAE.

Comment #4: An alternative experimental approach to support the claim that "improved recovery was the result of increased T cell apoptosis after activation in the CNS" would be to use genetically engineered T cells that are resistant to apoptosis in the CD4ΔAhr mice and demonstrate that these T cells are resistant to metabolite-induced apoptosis and that the remission phenotype is lost.
Answer: This is an excellent suggestion but based on the time constraints of resubmission at PLOS BIOLOGY (3 months) we were unable to do the proposed experiment and selected to administer taurocholic acid (Figure 6 G-I).

Comment #5: Figure 2A-K: There are no differences clinical outcomes in co-housed mice, so it is unclear to this reviewer why the authors used these groups to analyze potential differences in T cell skewing and T cell numbers in MLN, PP and iLN. Are these results similar in separately housed groups?
Answer: **The separately housed data are presented in Figure S2 and show the same results as Figure 2.**

Comment #6: Other comments related to Figure 2A-K:
- It would help readability and interpretation if the difference between co-housed vs separately housed cohorts were clearly labeled in the figure (not just in legends). Options authors could consider include the use of different color schemes, or notations ie Ahrfl/fl-CH vs CD4creAhrfl/fl-CH and Ahrfl/fl-SH vs CD4creAhrfl/fl-SH.

Answer: **Notations were added to the Figure.**
- Figure Legends and text describing the T cell skewing assays need to be clarified. It is currently unclear how many biological vs technical replicates were performed. (Is the data showing 6-8 individual mice with single replicates? Were all skewing assays performed on the same day or across multiple days?)

Answer: In our manuscript, "n" refers to the total number of individual mice per group. All are displayed in the figure unless otherwise noted. "N" refers to the number of separate experiments that were completed on separate days. For the skewing experiments, experiments were replicated twice or thrice on separate dates with a total of 6-8 mice per group across those experiments. This has been clarified in the legends.
- Methods also need more details: cytokine and antibody conditions for skewing assays, time of culture, any manipulation prior to collecting supernatants for cytokine quantification, etc. This should be in addition to the existing citation to previous lab papers.

Answer: Methods have been expanded to include more details.
- Can authors provide some representative flow plots for readers to evaluate gating of transcription factors?

Answer: Gating strategies have been added to figure 2

Comment #7: Figure 2L-Q: In these panels, the authors present total cytokine expression in spinal cord homogenates taken at peak disease in separately housed mice and conclude that "these data reinforce the hypothesis that the T cell-intrinsic reduction of Ahr activity in Cd4creAhrfl/fl mice is not responsible for EAE recovery". This may be an overinterpretation given that the analysis is not CD4 intrinsic and that the data appears underpowered (n=3, 1 repeat) to detect potential differences (TNFα looks especially intriguing). At minimum, this analysis should be repeated with a larger cohort. Flow cytometric measurement of intracellular cytokine staining could be even more informative.
Answer: We have repeated the Luminex assay as requested by the reviewer. We did not have enough mice ready to repeat the flow cytometry experiment before the deadline for resubmission.

Comment #8: Figure 3: Authors compare total numbers of CD4, CD8, RORγt, Foxp3, GATA3, and Tbet-expressing cells at peak and chronic phases, identifying diminished numbers of CD4+ T

cells in the SC of CD4ΔAhr mice at late stages (and CD11b at peak, but not chronic stages). A few extra analyses could really increase the clarity of what is happening here.

- Can authors clarify whether the RORγt, Foxp3, GATA3, and Tbet-expressing cells are pre-gated on CD4+ T cells?

Answer: Gating strategies have been added (see figure 3G).

- Are there any differences in the proportion of different T helper subtypes? For example, pre-gating on CD4+ T cells and then showing the frequency of Tbet vs RORγt-expressing cells could indicate whether there are defects in Th1 vs Th17 polarization.

Answer: We did not detect any differences in the proportions of each T-cells subtypes. They are equal across the board.

- Can authors include the CD11b+ quantification at both time points?

Answer: Our revision includes a quantification of CD11b expression by IHC staining in the spinal cord slices at peak and chronic phase of EAE (Fig 3 E-F). Our new results show no difference in the number of CD11b cells confirming our flow cytometry data.

- In Figure 2, the authors suggest there are no differences in cytokine expression in the SC homogenate at peak disease. Demonstrating a difference in cytokine expression (either via SC homogenate or intracellular cytokine staining) could support the hypothesis that the activity of effector CD4+ T cells is diminished in CD4ΔAhr mice.

Answer: Our data do not support the hypothesis that AHR deficiency in T cells change cytokine expression in the EAE spinal cord. Therefore, we attribute our phenotype to a decreased viability of T-cells rather than cytokine production capacity.

- Fig 3E & F both appear to have been only performed once and should be repeated if this is the case.

Answer: Unfortunately, during the revision process our mice did not breed enough to complete this experiment. As an alternate approach, quantification of CD11b expression by IHC staining in the spinal cord slices at peak and chronic phase of EAE is now presented (Fig 3 E-F). Our new results show no difference in the number of CD11b cells confirming our flow cytometry data.

- Does flow data of CD45 and CD3 staining corroborate immunohistochemistry staining shown in Fig 3B & D?

Answer: Flow data does corroborate Fig 3B and D, but are not presented in the manuscript.

Comment#9: Figure 4: authors identify increased apoptosis in CD4+ cells of the CD4ΔAhr mice.

- Please provide information on the samples used the gene expression data in Fig 4A ie numbers of mice, were samples pooled or analysed separately, was the analysis done once or repeated? If done only once, the data would benefit from validation in a separate cohort using targeted expression analysis of genes of interest.

Answer: This low throughput QCR array was initially done to discover pathway(s) modulated by AHR deficiency (Fig S3A). This gene expression dataset allowed us to highlight the role of AHR in T cells viability/apoptosis that were confirmed in Figures 2 and S3B-D.

- Please provide representative flow plots to show viability gating from SC and in vitro assays (could be in supplement if necessary). If available, fluorescence minus one (FMO) controls would be welcome.

Answer: This has been added to supplement S3.
- Text states that myeloid cell viability was tested, but this is not shown in Fig 4D.

Answer: This was a typo and has been revised to specify total TCRB cells.
- Please provide more details on culture conditions with the cecal contents. When were the filtered <3kDa molecules added? If present from the beginning, was there any effect on Th17 polarization or proliferation, or is this a selective survival effect?

Answer: Details were added in methods and results sections. In short, T cells were treated after differentiation during the 24-hour stimulation period. We also conducted some pilot experiments using them during skewing and stimulation (4-5 day) but noted toxicity with the dose of cecal metabolites selected.

Comment #10: Figure 5: The text mentions that there were "six primary or secondary bile acids significantly increased in the cecal microenvironment of Cd4creAhrfl/fl mice". Eight are shown in the associated figure. Can the authors specify the six in the text (and perhaps provide graphs for each)?

Answer: The bile acids have been added to text. Also in the text, authors state that deoxycholic acid can be used as a proxy for changes in microbial activity - was there any difference in DCA or ratio of primary: secondary bile acids in CD4ΔAhr mice?

Answer: There were no detected differences in our mice so we have moved this to the discussion to avoid confusion for readers.

Comment #11: It may be worth adding a discussion of how the authors think that cecal metabolites could be linked to T cell apoptosis in the spinal cord. There is some mention that bile acids and SCFA can be found in the SC, is this the most likely explanation, or are there other possibilities worth mentioning? Is it possible circulating metabolites could gain access to the LN to influence cell viability during priming, or that circulating metabolites could impact cells migrating from the LN to the CNS?

Answer: This has been added to discussion. We believe, based on previous literature showing that T cells traffic to the gut before entering the CNS during EAE, that they are being primed in the presence of these factors which is leading to apoptosis after they have migrated to the CNS. Future studies will be aimed at discovering the site of action of bile salts.

Comment #12: Discussion paragraph 3 (line 356-366) focuses on ways the adaptive immune system could be modulating the microbiome. The changes mentioned (IgA and AMP) might have more effect on shaping the communities, although there is little effect of the CD4-Ahr deficiency on community composition (at least at the taxonomic level shown). Could authors speculate on other mechanisms that could influence community function?

Answer: New data addressing how bile acids may be modulated were added to Figure 5 and subsequent discussion of bile acids has been included.

Minor comments:

* Intro line 69-70: question the authors are asking isn't very clear on first reading, consider

revising (state explicitly that you're asking whether Ahr expression influences microbiome composition and/or function).
Answer: This sentence has been revised.

* Could a CD4cre also be acting non-specifically? In particular, populations of gut macrophages express CD4 and thus are located at the site where these metabolites are present. Some discussion of other CD4-expressing cells that could play contribute to interactions between the immune system and the microbiome may be warranted.

Answer: Discussion has been expanded to include this potential explanation.

* Figure 1: Can authors provide information on the number of mice the images are representative of?

Answer: The number of mice (n) is now listed in Figure legend 1c and 1f

* Figure legends include descriptions of the results. Legends should be re-written to focus on key experimental details.

Answer: The figure legends have been updated.

* It's not clear that a 2-way ANOVA is the most appropriate statistical test for quantifying the # of CD45+ or CD3+ cells in the different sections of the SC. Could authors simply sum the totals from each section and do a t-test between the genotypes?

Answer: We believe that 2-way ANOVA is adequate here, to quantify potential important changes affecting the whole spinal cord. In this way we can highlight the effects in the caudal spinal cord where most of the damage occurs in this model.

* Error in figure legend in supplemental Figure 2E-F. Data show cecum, but the legend suggests these data are from feces**.**

Answer: This error has been fixed.

Methods

- How were statistical outliers determined?

Answer: Description of outlier calculations is now included in the methods (ROUT method). All outliers are included in the raw data file supplement Data S1 (labeled in blue) but excluded in the main figures of the manuscript.

- Can authors clarify the statement about animals that did not develop signs of EAE being excluded from analysis - were they also excluded from the incidence curves shown in the supplement? If so, perhaps authors can transform these data into 'Day of Onset' charts as incidence should include resistant mice.

Answer: Mice that did not develop EAE were included in incidence curves, but not in clinical score curves. Statement in methods added to clarify.

- Microbiome transfer is listed but not performed

Answer: It has now been performed and is data is included in figure 6.

- For T cell skewing assays - can authors provide more details. How many mice (biological replicates), how many technical replicates? Brief description of skewing media in addition to citation.

Answer: The information requested have been added to the manuscript.

- When were metabolites added to the in vitro skew, and how does that impact interpretation?

Answer: The information requested have been added to the methods. The metabolites were added after the cells were differentiated during the 24-hour stimulation period. With this approach, we can show acute effects and detect early apoptosis. This result complements the data from separately housed animals which demonstrate an AHR dependent apoptosis phenotype.

Experimental procedures mentioned in text but not described in Methods:
Luminex
Answer: The information requested have been added to the manuscript.
LFB/H&E, methods for quantifying % myelin coverage**.**
Answer: The information requested have been added to the manuscript.
The text for the Immunohistochemistry and Fluorescent Microscopy section needs to be edited for clarity.
Answer: The manuscript has been modified to improve clarity.

Reviewer #2:
Comment #1: Overall, the authors present an interesting work focusing on AHR and T cell biology in context of microbial metabolomics. However, the authors "only" provide an interesting link without any further in vivo validation, which would definitely boost the data. I would strongly suggest to validate the findings using microbial transfer studies or dietary studies supplementing bile acids or SCFA. If the authors can provide further experiments in vivo, I would recommend the paper for publishing.
Answer: To address this concern, we have therapeutically administered taurocholic acid (or vehicle) to mice undergoing EAE (Figure 6 G-I). Our new data shows that this treatment is protective in EAE and confirm other studies showing that other bile acids and SCFA can be protective in this model (Ho PP, Steinman L. Obeticholic acid, a synthetic bile acid agonist of the farnesoid X receptor, attenuates experimental autoimmune encephalomyelitis. Proc Natl Acad Sci U S A. 2016 Feb 9;113(6):1600 and Lewis ND, Patnaude LA, Pelletier J, Souza DJ, Lukas SM, King FJ, et al. A GPBAR1 (TGR5) Small Molecule Agonist Shows Specific Inhibitory Effects on Myeloid Cell Activation In Vitro and Reduces Experimental Autoimmune Encephalitis (EAE) In Vivo. PLoS One. 2014 Jun 26;9(6):e100883.)

Comment #2: The section header for the first results section is misleading. No gut microbiota composition was analyzed, therefore, the authors can not state that the gut flora is the reason for the observed differences. I strongly recommend to change the heading towards a more appropriate title.
Answer: Changed to "CD4+ specific *Ahr* knockout mice recover from EAE only when housed separately".

Comment #3: "Apoptosis of Ahr deficient CD4 T cell is microbiome dependent" - here as well. I would be more specific since you hypothesize that microbial metabolites are to reason. Please choose a more appropriate title.

Answer: Changed to "Apoptosis of *Ahr* deficient CD4 T cells is dependent on gut-derived small molecules".

Comment #4: Please provide representative histograms or dot plots for the flow cytometry experiments within the figure.
Answer: Included in relevant figures.

Comment #5: Line 86-90: redundant information as previously described in the introduction. Please remove.
Answer: Removed.
Comment #6: What was the rationale for conducting the 16s seq. in different facilities?
Answer: Purely logistical as 16S sequencing runs were done during the COVID pandemic and we had to change providers due supply chain issues and availability of services.

<u>**Reviewer #3:**</u>

Comment #1: I would encourage the authors to revisit the vocabulary used in the paper. Quite often, the authors use vocabulary not suited for the field of immunology. Editing by an immunologist will help to solve this issue.
Answer: Thank you for this note, our manuscript was edited by our immunologist colleagues to improve the language.

Comment #2: Multiple comparisons need to be preceded by ANOVA or Kruskal Wallis test.
Answer: All multiple comparisons are preceded by ANOVA (two-way or mixed effects) or Kruskal Wallis test as indicated in the text.

Comment #3: "While most of the canonical immune sensors respond exclusively to pathogenic material…..". This is not true, as most of the immune receptors have endogenous ligands.
Answer: Revised to "While canonical immune sensors have been primarily characterized based on their response to pathogenic material, the AHR is a homeostatic regulator…"

Comment #4: Line 95, 105, and thereafter "..myeline coverage…". This is an odd way of putting it. Usually, people refer to "myelin content", "amount of myelin".
Answer: Given that LFB is a histological stain, we have updated figures to read "% Myelin area" and we have revised to "myelin staining" in the text.

Comment #5: ".. active EAE scores" do the authors mean "clinical scores"?
Answer: Changed to "clinical scores" throughout.

Comment #5: "Representative plot 111 includes n= 9/group; N=2". What is n 9 or 2?
Answer: "n" indicates the number of total number of animals per group shown in the representative graph. "N" signifies that this experiment was completed twice with similar results. More details were added to legends

Comment #6: Although the authors correctly use non-parametric statistics comparing clinical scores, the Mann-Whitney U test is used for two groups comparisons. Adding another dimension, such as time, requires the Kruskal Wallis test.
Answer: Based on the following citation, Mann-Whitney U test would be more appropriate for our analyses: (Kandace K. Fleming, James A. Bovaird, Michael C. Mosier, Mitchell R. Emerson, Steven M. LeVine, Janet G. Marquis; Statistical analysis of data from studies on experimental autoimmune encephalomyelitis; Journal of Neuroimmunology; Volume 170, Issues 1–2,2005,Pages 71-84, ISSN 01655728,https://doi.org/10.1016/j.jneuroim.2005.08.020

Comment #7: If the authors used H&E coupled with luxol fast blue, how did they quantify the area of myelin? Usually, for quantification, luxol fast blue is done separately so that amount of stained tissue can be quantified.
Answer: Quantification was done with luxol fast blue alone but luxol fast blue with H&E are shown in the figure to give a better representation of lesions. Figure 1 legend modified to make this clear.

Comment #8: Fig 1 F, For comparisons of myelin content, ANOVA will be more appropriate. Was there a difference between KO and WT at the chronic stage?
Answer: We have selected a t test to assess the increase of myelin (remyelination) between the peak and the chronic phase in AHR deficient mice. For clarity, we have separated these data into two graphs. For your information, an ANOVA results in a p value of 0.1574 and no difference between KO and WT at the chronic stage.

Comment #9: "T cell skewing activity" is not the correct term. Differentiation of T cells can be skewed towards different phenotypes.
Answer: Text has been revised to say "T cell differentiation and activity…"

Comment #9: In figure 4, the authors propose that activation-induced cell death is increased in TH17 cells from KO mice. The authors need to acknowledge that anti-CD3 activation is not considered physiological. Most commonly, anti-CD28/CD3 is used.
Answer: While anti-CD3/CD28 restimulation is more accepted; stimulation with anti CD3 has also been reported (https://translational-medicine.biomedcentral.com/articles/10.1186/1479-5876-8-104). We acknowledge this limitation in the text.

Comment #10: Line 394 In this way, we have two independent experiments resulting in appropriate power. What is statistical power in these experiments?
Answer: For each test the power is 0.8 or higher.

Comment #11 Line 399 "Statistical outliers are not included in analysis or figures" How were outliers determined? How many were excluded?
Answer: Description of outlier calculations included in the methods (ROUT method). All outliers are included in the raw data file supplement Data S1 but excluded in the main figures of the manuscript.
