# Supplementary material for: The activity of the aryl hydrocarbon receptor in T cells tunes the gut microenvironment to sustain autoimmunity and neuroinflammation
Source: PLoS Biol. 2023 Feb 14;21(2):e3002000. doi: 10.1371/journal.pbio.3002000 (PMC9928083; doi:10.1371/journal.pbio.3002000)
Supplement: Attachment — Submitted filename: Response to Editors.pdf [file pbio.3002000.s009.pdf]

The sentence in the discussion in response to Reviewer #1 to acknowledge the possibility that observations in the CD4-cre mice could reflect CD4+ macrophages should be revised to say that you can't rule out a role of AHR in other cell types developmentally affected by CD4-expression including CD8 T cells. CD4 is expressed in double positive T cells during thymic development, so AHR would be deleted in both CD4 and CD8 T cells.

This information was added at lines 389-391 in order to fit with the flow of the paragraph.

1) Supplementary files (e.g., excel). Please ensure that all data files are uploaded as 'Supporting Information' and are invariably referred to (in the manuscript, figure legends, and the Description field when uploading your files) using the following format verbatim: S1 Data, S2 Data, etc. Multiple panels of a single or even several figures can be included as multiple sheets in one excel file that is saved using exactly the following convention: S1_Data.xlsx (using an underscore).

Done throughout.

2) Deposition in a publicly available repository. Please also provide the accession code or a reviewer link so that we may view your data before publication.

The datasets supporting the conclusions of this article are available in the NCBI Gene Expression Omnibus repository accession number GSE200440

https://www.ncbi.nlm.nih.gov/geo/query/acc.cgi?acc=GSE200440

Regardless of the method selected, please ensure that you provide the individual numerical values that underlie the summary data displayed in the following figure panels as they are essential for readers to assess your analysis and to reproduce it: Figures 1ACDFG, 2BCDEFGHIKLMNOPQRS, 3ABCDEFHI, 4BCDEF, 5BCDE, 6BCEFHI, and supplementary figures S1ABCDEF, S2ABCDEFG, S3ABCD, S4ABCDEF.

This is included in S1 Data with the exceptions of 5BC (which is in S2 Data and S3 Data) and Supplement 4 (which is deposited in Omnibus). These are all noted in the legends.

Please also ensure that figure legends in your manuscript include information on where the underlying data can be found, and ensure your supplemental data file/s has a legend.
At the end of each legend, this information is included. Supplemental data file legends listed at the end of the submitted manuscript document.

2. Please provide a blurb which (if accepted) will be included in our weekly and monthly Electronic Table of Contents, sent out to readers of PLOS Biology, and may be used to promote your article in social media. The blurb should be about 30-40 words long and is subject to editorial changes. It

should, without exaggeration, entice people to read your manuscript. It should not be redundant with the title and should not contain acronyms or abbreviations.

We deleted the aryl hydrocarbon receptor, a sensor for the gut microbiota, in T lymphocytes- initiating a change in intestinal bile acid and short chain fatty acid composition. These metabolites arise as putative novel protective agents in multiple sclerosis.

3. We suggest a modification in the title to make it more accessible: "The activity of the aryl hydrocarbon receptor in T cells tunes the gut microenvironment to sustain autoimmunity and neuroinflammation".

Title changed
